# Efficacy of Fuzheng Quxie Formula Against Postoperative Metastasis of Lung Cancer in Stage IIA-IIIA With Negative Driver Genes: Protocol for a Multicenter, Double-Blind, Randomized Controlled Trial

**DOI:** 10.2196/66342

**Published:** 2025-10-24

**Authors:** Fan Xu, Yun Yang, Yinbin Luo, Bin Luo, Jialiang Yao, Yiyang Zhou, Minghua Li, Jianchun Wu, Wenfei Shi, Lei Jiang, Qian Huang, Wentao Fang, Zhihong Fang, Yan Li, Jianhui Tian

**Affiliations:** 1 Clinical Oncology Center, Shanghai Municipal Hospital of Traditional Chinese Medicine, Shanghai University of Traditional Chinese Medicine Shanghai China; 2 Department of Thoracic Surgery, Shanghai Lung Hospital of Tongji University Shanghai China; 3 Cancer Center, Shanghai General Hospital, Shanghai Jiao Tong University School of Medicine Shanghai China; 4 Department of Thoracic Surgery, Shanghai Chest Hospital, Shanghai Jiao Tong University School of Medicine Shanghai China

**Keywords:** traditional Chinese medicine, Fuzheng Quxie Formula, lung cancer metastasis, negative driver genes expression, efficacy, safety

## Abstract

**Background:**

Metastasis is the primary cause of poor prognosis and high mortality in lung cancer. Surgery with postoperative adjuvant chemotherapy is the standard treatment for patients with stage IIA-IIIA lung cancer with negative driver genes. However, recurrence rates remain significant. In China, traditional Chinese medicine shows potential as an adjuvant therapy to reduce treatment toxicity and improve clinical efficacy.

**Objective:**

This study aimed to evaluate its efficacy and safety in preventing postoperative metastasis in driver gene-negative stage IIA-IIIA lung cancer, based on the promising preclinical results of Fuzheng Quxie Formula against lung cancer metastasis. In this trial, we hypothesize that the treatment group will have better efficacy and safety than the control group.

**Methods:**

A multicenter, double-blind, randomized, placebo-controlled parallel group trial will be conducted. Eligible patients will be randomized into a treatment group (daily Fuzheng Quxie Formula granules+regular chemotherapy) and a control group (daily Chinese medicine placebo granules+regular chemotherapy) in a ratio of 1:1. Fuzheng Quxie Formula will be administered orally, twice a day, in the morning and evening, for 6 months. Patients will be followed up after the end of treatment for 18 months. After the end of the program, follow-up will be continued for 5 years or until the patient dies (or progressed). The primary efficacy endpoint is disease-free survival, and the secondary efficacy endpoints are overall survival, minimal residual disease, circulating tumor cells, Chinese medicine symptom score, quality-of-life assessment, immune indicators, tumor markers, peripheral blood systemic immune-inflammation index, and prognostic nutritional index. We will conduct per-protocol analyses on these outcomes. In addition, we will also evaluate the safety of the Fuzheng Quxie Formula.

**Results:**

This study began screening and recruitment in March 2023. Recruitment is ongoing; by the end of 2024, a total of 180 eligible participants will be enrolled. Recruitment will continue until the end of June 2025 or until the target sample is reached. We estimate that the results will be published by March 2026.

**Conclusions:**

This study is a high-quality, large-scale, multicenter, double-blind, randomized controlled trial. This will be the first trial to evaluate the efficacy and safety of Fuzheng Quxie Formula in inhibiting metastasis after surgery in stage IIA-IIIA lung cancer with negative driver genes. Provide a basis for the clinical application of Fuzheng Quxie Formula.

**Trial Registration:**

ClinicalTrials.gov NCT06381960; https://clinicaltrials.gov/study/NCT06381960

**International Registered Report Identifier (IRRID):**

DERR1-10.2196/66342

## Introduction

### Background and Rationale

Lung cancer is one of the most common malignant tumors of the respiratory system, with the mortality rate perennially ranking first among all kinds of malignant tumors, which greatly threatens the safety of human life [1]. In developed countries (such as the United States), lung cancer is still the malignant tumor with the highest number of deaths [2]. In China, the latest data shows that there are about 787,000 new cases of lung cancer, with an incidence rate of 57.26/100,000, ranking first in the national morbidity and cause of death by gender, causing economic losses of up to 221.4 billion, and this economic and social pressure will continue to increase [3].

International guidelines for the diagnosis and treatment of lung cancer recommend that radical surgery is the first choice for stage I-IIIA non–small cell lung cancer (NSCLC). Stage IA NSCLC does not require postoperative adjuvant chemotherapy, while stage IB (high-risk) NSCLC could consider receiving postoperative adjuvant chemotherapy. Stages IIA to IIIA NSCLC must undergo postoperative adjuvant chemotherapy. Stages IIIB-IV NSCLC are treated with nonsurgical therapy as the standard first-line treatment [4]. Stage IIA-IIIA NSCLC has a moderate to high risk of recurrence [5]. Studies have shown that the 5-year recurrence rate for patients with stage I lung cancer after adjuvant chemotherapy is approximately 17.8%, while approximately 45.5% of patients with stage II-III lung cancer may experience metastatic recurrence within 5 years [6]. Data from a survival follow-up study of 7753 patients with lung cancer surgery conducted by Wang Zhezhou’s team showed that the 5-year survival rates for patients with stage IIA, IIB, and IIIA lung cancer were 78.2%, 62.9%, and 49.3%, respectively. As such, postoperative standard chemotherapy for patients with stage IIA-IIIA lung cancer demonstrates limited efficacy in preventing recurrence and metastasis following curative surgery. In addition, chemotherapy is associated with significant toxicity, making it difficult for many patients—particularly the older adults and frail—to tolerate or accept. Therefore, postoperative standard chemotherapy exhibits overall poor efficacy in preventing recurrence and metastasis following curative lung cancer surgery in stage IIA-IIIA. With the continuous advancement of targeted therapy and immunotherapy, some patient groups have benefited from these treatments, and the clinical treatment landscape is gradually improving. However, an urgent clinical challenge remains, and patients with negative driver genes and extremely low PD-1 (programmed death-1)/L1 (PD-1 ligand) expression (1%) often fail to achieve optimal therapeutic outcomes with current treatment modalities. There is an urgent need to develop effective treatment strategies to prevent postoperative metastasis in patients with lung cancer at stage IIA-IIIA who are negative for driver genes.

Traditional Chinese medicine has a long history of application in the prevention and treatment of tumors. Through long-term clinical practice, it has accumulated and developed a systematic theoretical framework and rich experience. Currently, its advantages in adjuvant chemotherapy have been recognized internationally—numerous studies have confirmed that traditional Chinese medicine offers multiple benefits in adjuvant chemotherapy, including reducing toxicity [7,8] and enhancing efficacy [9], combating chemotherapy resistance [10], regulating immunity, and improving quality of life and treatment tolerance [11].

Professor Jianhui Tian's group focuses on lung cancer metastasis research. Under the guidance of a master of Chinese medicine Liu Jiaxiang's academic idea of “treating cancer by reinforcing healthy qi,” and incorporating Lao Zi’s “Existence and nonexistence give birth the one to the other,” modern oncology and immunology, professor Jianhui Tian's group suggests the core pathogenesis of cancer metastasis in the subclinical stage of cancer —“Deficiency of Vital Qi and Hidden Toxin” theory. According to the theory, immune disorders caused by immune senescence and immune escape are “Deficiency of Vital Qi,” while circulating tumor cells, quiescent cancer cells, and tumor stem cells are the modern biological connotations of “hidden toxicity.” The residual cancer cells after radical treatment of lung cancer are the “Hidden Toxin.” After radical lung cancer surgery, residual cancer cells (hidden toxin) are latent in the body and in a dormant or quiescent stage. When there is a deficiency of vital qi, the efficiency of immune surveillance and immune clearance decreases, and the cancer cells go from the quiescent phase to the proliferative phase, leading to the occurrence of tumor metastasis. Professor Jianhui Tian’s group further found that myeloid-derived suppressor cells, regulatory T cells, and circulating tumor cells (“hidden toxin”) are important factors in the subclinical state of lung cancer metastasis in clinical studies, and analyzed their expression patterns. The group took the lead in establishing the world’s first human lung adenocarcinoma circulating tumor cell line (CTC-TJH-01), as well as a platform for metastasis-specific studies of lung cancer.

Under the theory of “Deficiency of Vital Qi and Hidden Toxin,” the group established the treatment method of “supporting vital qi” and “dispelling hidden toxin,” and created a Chinese medicine formula, “Fuzheng Quxie Formula,” for inhibiting postoperative metastasis of early- and middle-stage NSCLC. In this formula, Sheng Huang Qi, Fu Ling, Bai Zhu tonify spleen and stomach to “strengthen earth to generate metal;” Bei Sha Shen, Tian Dong, Mai Dong nourishes yin and moistens the lungs; Hai Zao, Xia Ku Cao, Kun Bu, which soften and resolve hard mass, and Bai Hua She She Cao, Shi Jian Chaun, Shi Shang Bai, which clear heat, detoxifying to disperse nodules. This formula is primarily indicated for patients with lung cancer presenting with clinical manifestations of qi and yin deficiency syndrome. The specific diagnostic criteria for qi and yin deficiency syndrome are detailed in the inclusion criteria section. Through in-depth clinical and basic research, the team has obtained the scientific basis for Fuzheng Quxie Formula to inhibit metastasis: (1) Fuzheng Quxie Formula has the clinical effect of preventing recurrence and metastasis: the group, in cooperation with Shanghai Lung Hospital, carried out a prospective clinical study, which confirms that the disease-free survival (DFS) rates of early- and middle-stage patients with NSCLC after intervention with Fuzheng Quxie Formula were 98.8% and 96.5% in 1 and 2 years, respectively, which were higher than 95.3% of the patients in the pure follow-up group. (2) The mechanism of inhibiting metastasis by Fuzheng Quxie Formula may be to regulate the immune function to promote apoptosis of circulating tumor cells: intragroup comparison of immune indexes before and after the study suggests that the peripheral blood natural killer, CD3^+^ T cells and IgM content of the treatment group after intervention of Fuzheng Quxie Formula increased significantly (*P*<.01), and at the same time, it can reduce the number of circulating tumor cells in the blood (*P*<.01), suggesting that Fuzheng Quxie Formula has the tendency to regulate patients’ immunity and inhibit circulating tumor cells to control tumor progression; (3) mechanism study found that Fuzheng Quxie Formula can improve immune function and restore the state of body immune balance: Fuzheng Quxie Formula can up-regulate the expression of positive immune cells, such as natural killer cells and T cell subpopulations, which have an inhibitory and killing effect on tumor cells, and down-regulate positive immune cell expressions, which promote tumor cell proliferation, metastasis, immune escape, and so forth, downregulate the expression of negative immune cells such as myeloid-derived suppressor cells and regulatory T cells, which promote the proliferation, metastasis and immune escape of tumor cells; inhibit and remove circulating tumor cells in the peripheral circulation; and influence and regulate the metabolic reprogramming of tumor cells in terms of sugar, fat and amino acids. Meanwhile, the theory of “Deficiency of Vital Qi and Hidden Toxin” guided the construction of clinical efficacy prediction and metastasis risk assessment model for traditional Chinese medicine (TCM) treatment of lung cancer, which found that maintaining peripheral immune homeostasis is an important basis for TCM to inhibit lung cancer metastasis, and optimized and enriched the prognostic assessment system of lung cancer.

Fuzheng Quxie Formula has been applied as an in-hospital preparation for many years and has successfully entered the process of joint development of new drugs with enterprises. Therefore, in response to the urgent clinical needs of postoperative patients with IIA-IIIA NSCLC who are negative for driver gene expression, we attempted to objectively evaluate the efficacy of Fuzheng Quxie Formula in inhibiting metastasis of IIA-IIIA NSCLC who are negative for driver gene expression and safety.

### Objectives

This study will be the first trial to evaluate the efficacy and safety of Fuzheng Quxie Formula in inhibiting metastasis after surgery in stage IIA-IIIA lung cancer with negative driver genes.

#### Primary Objective

The primary objective of this study was to demonstrate that the use of Fuzheng Quxie Formula can prolong the DFS of patients with negative driver genes in stage IIA to IIIA lung cancer.

#### Secondary Objectives

Comparing the overall survival (OS), minimal residual disease (MRD), circulating tumor cell assay, Chinese medicine symptom score, quality-of-life assessment, immune indicators, tumor markers, systemic immune-inflammation index (SII), and prognostic nutritional index (PNI) between the 2 groups of patients. Further evaluate the clinical efficacy of the tonifying and detoxifying formula in the prevention and treatment of lung cancer metastasis, and simultaneously verify its safety in application.

## Methods

### Trial Design

**Figure 1:**
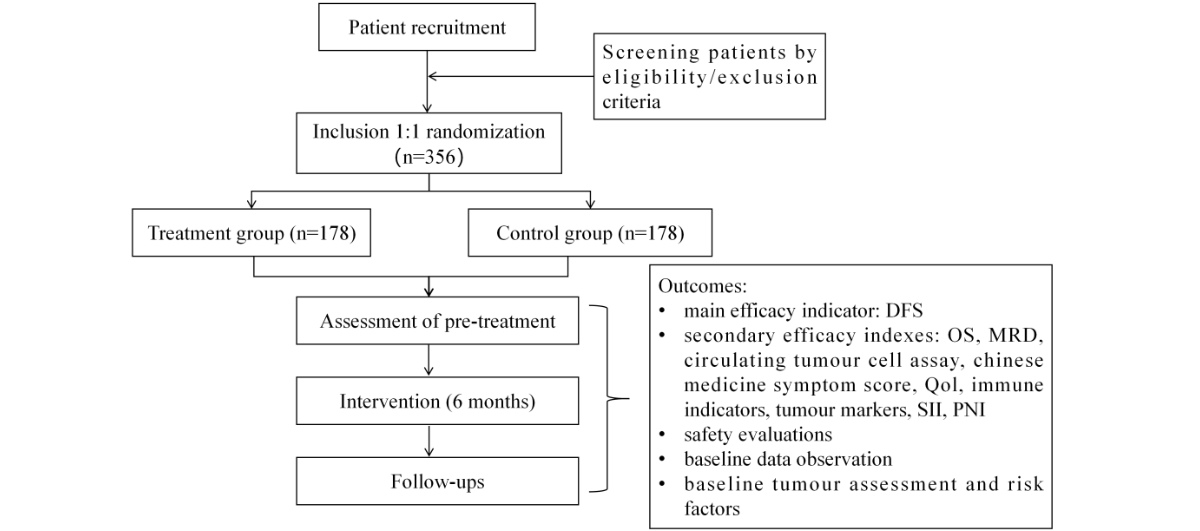
Study flowchart. DFS: disease-free survival; OS: overall survival; MRD: minimal residual disease; SII: systemic immune-inflammation index; NSCLC: non–small cell lung cancer; PNI: prognostic nutritional index; QoL: quality-of-life assessment.

A prospective clinical study of combined Chinese and Western medicine for the prevention and treatment of metastasis after radical lung adenocarcinoma in patients with postoperative stage IIA-IIIA NSCLC adenocarcinoma with negative driver gene expression is being conducted in a randomized, double-blind, placebo-parallel controlled, multicenter clinical trial design (Figure 1). First, the clinical study will obtain written informed consent from the participants. Eligible participants will be randomly assigned in a 1:1 ratio to the treatment group (daily Fuzheng Quxie Formula granules+regular chemotherapy) and the control group (daily Chinese medicine placebo granules+regular chemotherapy). The TCM treatment period is 6 months, with follow-up at the end of the period, which is 18 months. After the end of the program, follow-up will continue for 5 years or until the participant dies (or progresses).

### Sample Size

The calculation of the sample size of this clinical trial is based on the statistical results of the “Chinese Medicine Clinical Research on Common Malignant Tumors (Lung Cancer)” conducted by this group in the third round of the Shanghai 3-year action plan of traditional Chinese medicine. Based on the group’s previous clinical research on the survival and immune function effects on patients after radical NSCLC surgery, the 5-year DFS rate was 90.2% in the treatment group and 78.3% in the control group. Sample size was calculated using PASS 15 software (NCSS, LLC), and survival-2 survival curves-test (inequality)-log-rank tests were applied. The values were set as follows: power=0.8, α=0.025 (unilateral), according to the control group and experimental group 1:1, combined with the basis of the previous research, taking the proportion surviving as input type, S1 (proportion surviving: control)=0.783, S2 (proportion surviving: treatment)=0.90, T0 (survival time)=5 years, accrual time (integers only)=2 years, uniform enrollment, the rate of shedding in each group is 10%, the calculation can be calculated to obtain a total sample size of 317 cases, 158 cases in the treatment group and 159 cases in the control group. At the same time, there are 5 clinical trial centers in this study. According to the sample size estimation requirements of multifactorial analysis, the introduction of one more confounding factor needs to increase the sample size of each group by 15-25 cases, and this study considers an increase of 20 cases in each group. Therefore, 178 cases need to be collected in each group, and the total sample size of the 2 groups is 356 cases (Table 1).

**Table 1:** Sample size estimation for each center.

Research center	Treatment group, n	Control group, n	Total, n
Shanghai Municipal Hospital of Traditional Chinese Medicine, Shanghai University of Traditional Chinese Medicine	38	38	76
Shanghai Lung Hospital of Tongji University	35	35	70
Shanghai Chest Hospital, Shanghai Jiao Tong University School of Medicine	35	35	70
Shanghai General Hospital, Shanghai Jiao Tong University School of Medicine	35	35	70
Shanghai Hospital of Integrated Traditional Chinese and Western Medicine, Shanghai University of Traditional Chinese Medicine	35	35	70
Total	178	178	356

### Assignment of Interventions: Allocation

#### Sequence Generation

The stratified random grouping scheme used in this study was prepared by a statistician. The entire study population that met the inclusion criteria was allocated according to the stratified random grouping scheme. The sample size of each stratum was a multiple of the number of groups. The stratification factors were different subcenters. The randomization scheme was prepared by a teacher of medical statistics not related to the data management and statistical analysis of this trial, and a random table was generated by SPSS software (IBM Corp), with the treatment group: the control group in a ratio of 1:1 to generate a random code by using the method of zone group randomization.

#### Concealment Mechanism

The selected block lengths and randomized seed parameters are sealed together in a blinded base as confidential data. After blinding, the blinders of the statistical unit hand over the sealed blinds, including the primary and secondary blinds based on the randomization number, to the unit responsible for the clinical study, and the blinds are sealed in duplicate at the unit responsible for the clinical study.

#### Implementation

The entire study population that met the inclusion criteria was allocated according to the stratified random grouping scheme.

### Assignment of Interventions: Blinding

#### Who Will Be Blinded

This study was a double-blind trial, and neither the investigator nor the study population had access to subgroup information. Patient grouping is usually performed by third-party institutions or independent research teams. A 2-level blinded design was used, with the first level being the group corresponding to each case number (eg, group A or group B) and the second level being the treatment corresponding to each group (test or control). The randomized coding table was created by the statistical unit, and the 2 levels of blinded bottoms were individually sealed in duplicate and stored in the team leader’s unit and in the quality control office, respectively.

#### Procedure for Unblinding if Needed

When all the case report forms (CRFs) were entered into the database, and after questioning, verification, and blinding audit, the data were locked, and the first unveiling of blinding (ie, clarifying groups A and B) was carried out by the staff member who kept the blinded bottoms, which was handed over to biostatisticians to enter into the computer, and then statistically analyzed after linking up with the data files.

Blinding may be broken in emergency situations when the investigator believes that knowledge of the medication taken by the participant is beneficial for the management of adverse events. This should be done by the investigator, and a detailed record of the reason, time, and place of the blind breaking should be recorded and signed. Within 24 hours of the blinding, the responsible (lead) unit of the clinical trial and the clinical supervisor, as well as the statistically relevant personnel, should be notified, and the reason for the blinding should be explained. Once the contingency letter has been opened, the numbered participant will be withdrawn from the trial and treated as a dropout case, and the investigator should record the reason for withdrawal on the case report form. All contingency letters will be retrieved at the end of the trial, along with the case report form for blinded audit at the end of the trial.

### Participants

#### Recruitment

The sources of cases for this study were inpatients and outpatients admitted from March 1, 2023, to June 30, 2025, with negative expression of driver genes in postoperative stage IIA-IIIA lung adenocarcinomas with qi and yin deficiency. Inclusion for observation should be no later than 6 weeks postoperatively.

#### Study Setting

Participants were selected from 5 study centers: Shanghai Municipal Hospital of Traditional Chinese Medicine, Shanghai University of Traditional Chinese Medicine; Shanghai Lung Hospital of Tongji University; Shanghai Chest Hospital, Shanghai Jiao Tong University School of Medicine; Shanghai General Hospital, Shanghai Jiao Tong University School of Medicine; Shanghai Hospital of Integrated Traditional Chinese and Western Medicine, Shanghai University of Traditional Chinese Medicine.

#### Eligibility Criteria

In total, 356 patients will be included in the clinical trial by the research team members if they meet the study criteria. Screening will be based on inclusion and exclusion criteria (Textbox 1).

**Study inclusion and exclusion criteria.**

**Inclusion criteria**

- Patients with clinical staging of stage IIA-IIIA lung adenocarcinoma after radical surgery, within 6 weeks after surgery;
- Driver gene negative (no epidermal growth factor receptor, Anaplastic Lymphoma Kinase, ROS Proto-Oncogene 1 mutations), PD-1/L1 expression <1%;
- Fulfilling the diagnostic criteria for Qi-Yin deficiency syndrome, the primary symptoms are cough, little phlegm, shortness of breath, voice trailed off, weakness, and thirst without wanting to drink. Secondary symptoms include spontaneous sweating, night sweating, 5 upset hot symptoms, red tongue or teeth marks on the sides of the tongue, and a weak pulse. At least 2 primary symptoms and one of the secondary symptoms are present;
- Patients aged between 18 and 75 years;
- Patients with basically normal blood and biochemical indicators, without serious viral or bacterial infections; without organ failure and serious heart disease (blood bilirubin <68 μmol/L, aspartate aminotransferase<90 IU/L, creatinine <350 μmol/L, leukocyte count>3.5×109/L and <12×109/L, platelet count>80×109/L, erythrocyte pressure >0.20);
- Those with a tumor performance status score 2 and no other serious comorbidities;
- The patients themselves gave informed consent to participate in the study by signing an informed consent form with good compliance.
- Patients who are lactating and not pregnant;
- Passing the chemotherapy-related indicators;
- Those who have no allergic reaction to the ingredients in the formula.

**Exclusion criteria**

- Patients who are being treated with other drugs or therapies (including other Chinese herbal medicines, immunological drugs, radiotherapy, etc);
- Patients who are themselves mentally ill and have a lack of autonomous behavior;
- Women who are pregnant, preparing for pregnancy, or breastfeeding;
- Combined heart, lung, brain, liver, kidney, and hematopoietic system, and other serious diseases, and psychiatric patients;
- Allergic or known hypersensitivity to the components of the drug;
- Patients who are participating in other clinical trials or have participated in other clinical trials within 3 months;
- Alcohol or psychoactive substance abuse, drug abusers, and dependent persons;
- Other pathologies or conditions that, in the judgment of the investigator, have the effect of reducing the likelihood of enrolment or complicating enrolment, for example, frequent changes in the work environment, unstable living conditions, and other conditions that predispose to loss of visits.

#### Who Will Take Informed Consent?

They will be informed in detail about the content and precautions of this clinical trial before they decide whether or not to participate in this study. They will be informed that participation in the study is entirely voluntary and that they may withdraw from the study at any time during the treatment period without loss of follow-up medical care or other benefits, and that they will continue to be effectively treated by their doctor in accordance with normal medical procedures. If willing to participate in this study, they will sign an informed consent form.

#### Who Was Excluded After Joining the Trial?

Cases in which participants were found not to meet the inclusion criteria after enrollment; The use of prohibited medicines specified in the protocol (eg, other Chinese herbal medicinal tablets, immunotherapeutic drugs) needs to be excluded due to violation of the clinical trial protocol; cases that have not used the drug after inclusion need to be excluded; cases without any evaluable record after taking the drug. Reasons should be given for the exclusion of cases, and their CRF forms should be kept for reference. Those who will not be statistically analyzed for efficacy, but who have received at least one treatment with at least one safety record, may participate in the analysis of adverse reactions.

#### Dropout Criteria

A participant who meets the inclusion criteria but does not complete the trial for some reason is considered a dropout. It includes both cases where the participant withdraws on his or her own and cases where the investigator determines that the participant has withdrawn.

#### Conditions for Suspension of the Trial

Trial suspension refers to the stopping of a clinical trial in the middle of a trial that has not yet been completed as planned. The purpose of trial suspension is mainly to protect the rights and interests of the participants, ensure the quality of the trial, and avoid unnecessary economic losses. Discontinuation of the trial is required in the following cases: (1) serious safety problems occurred in the trial, the trial should be promptly suspended; (2) the trial found that the drug treatment effect is too poor, or even ineffective, does not have clinical value, the trial should be discontinued, on the one hand, to avoid delaying the effective treatment of participants, while avoiding unnecessary economic losses; if there is a disease progression or serious adverse reactions can be initiated by the withdrawal of the protection mechanism, that is, the inclusion of evaluable cases, the later assessment of efficacy data to invalid for carry-over. The lack or loss of efficacy is assessed by the investigator (not the participant). (3) Significant errors in the clinical trial protocol are identified during the trial, making it difficult to evaluate drug effects; or a well-designed protocol has undergone important deviations in its implementation, and further continuation makes it difficult to evaluate drug effects; (4) the sponsor requests suspension (eg, financial reasons and management reasons); and (5) withdrawal of the trial by the administrative authority, etc.

### Interventions

#### Trial Grouping

In the treatment group, patients received daily granules of Fuzheng Quxie Formula+regular chemotherapy, and in the control group, patients received daily Chinese medicine placebo granules+regular chemotherapy.

#### Trial Drug

The trial drug was Fuzheng Quxie Formula granules. Components of Fuzheng Quxie Formula granules are shown in Table 2. The drug was supplied by Jiangyin Tianjiang Pharmaceutical Co. All trial medications were provided as blinded and met quality requirements.

**Table 2:** Components of Fuzheng Quxie Formula.

Chinese name	Latin name	Parts of the substances	Amount (g)
Sheng Huang Qi	*Udis astragalus*	Root	15
Dang Shen	*Codonopsis pilosula*	Root	15
Jiao Bai Zhu	*Frixum atractylodes*	Root tuber	15
Bai Fu Ling	*Alba poria*	Root tuber	15
Bei Sha Shen	*Adenophora septentrionalis*	Root	15
Zhe Mai Dong	*Ophiopogon japonicus*	Root tuber	15
Hai Zao	*Alga*	Dried algae	15
Xia Ku Cao	*Prunella vulgaris*	Flowering fruit spike	15
Sheng Mu Li	*Udis ostreis*	Shell	15
Shi Jian Chuan	*Ishimi chuan*	Above-ground parts	15
Shi Shang Bai	*Cupressus lapis*	Whole herb	15
Shu Yang Quan	*Shuyang spring*	Whole herb	15

#### Explanation for the Choice of Comparators

Patients in the control group will receive placebo capsules in addition to chemotherapy. Since all study patients are allowed to be further treated with chemotherapy, there are no ethical concerns about this control condition. In total, 5% of the drug content of the treatment group was made into a control placebo drug, provided by Jiangyin Tianjiang Pharmaceutical Co, Ltd, which had the same odor, color, and appearance as the test drug, but had no significant drug treatment effect.

#### Method of Administration

Daily granules of Fuzheng Quxie Formula or daily Chinese medicine placebo granules are administered concurrently with regular chemotherapy.

Chemotherapy regimen selection will be selected according to National Comprehensive Cancer Network guidelines, and patients will be treated with platinum-based chemotherapy according to one of the following regimens for 3 weeks each for 4 cycles: (1) NP regimen includes vincristine, 25 mg/m^2^ on day 1, cisplatin, 75 mg/m^2^ on day 1; (2) GP regimen includes gemcitabine, 1250 mg/m^2^ on days 1 and 8, and 75 mg/m^2^ on day 1; (3) DP regimen includes docetaxel, 75 mg/m^2^ on day 1 and cisplatin, 75 mg/m^2^ on day 1; (4) AP regimen includes pemetrexed, 500 mg/m^2^ and cisplatin, 75 mg/m^2^ on day 1; and (5) TC regimen includes paclitaxel, 200 mg/m^2^ on day 1 and carboplatin, area under the curve 6 on day 1, and patients intolerant of cisplatin will be given area under the curve 5-6 on day 1, area under the curve 5-6 given carboplatin. All chemotherapeutic agents will be administered intravenously in both groups. The chemotherapeutic agents were administered strictly according to the appropriate instructions and National Comprehensive Cancer Network guidelines for recommended dosage, duration, and usage. These chemotherapy regimens are all recommended as equivalent standard chemotherapy regimens for first-line treatment of lung cancer. The core focus of this study is to evaluate the efficacy of adding a traditional Chinese medicine intervention to such standard chemotherapy. We allow research centers to select specific standard regimens (such as NP, GP, etc) based on routine practice to enhance the practicality and generalizability of the study.

For oral administration of traditional Chinese medicine, each bag was soaked in 400 mL of boiling water at 100 °C for 20 minutes, divided into 2 portions, each with a content of 200 mL. 200 mL was taken orally twice a day, in the morning and in the evening.

#### Test Period

The Chinese medicine treatment period is 6 months. The follow-up period is 18 months after the end of dosing. After the end of the program, follow-up will continue for 5 years or until the participant dies (or progresses).

#### Criteria for Discontinuing or Modifying Allocated Interventions

First, during the course of medication, if the participant develops an aggravation of the condition, he or she should discontinue the use of the test drug, complete the postevaluation of the efficacy and the relevant laboratory tests to end the trial, and the participant will be counted as an invalid eligible case for inclusion in the compliance with the protocol set (PPS). Second, if the investigator believes that other circumstances make it inappropriate for the participant to continue using the test drug.

#### Strategies to Improve Adherence to Interventions

We will provide medication to enrolled patients during the treatment period. During this period, the researcher will make regular follow-up visits.

#### Relevant Concomitant Care Permitted or Prohibited During the Trial

On the one hand, during the trial, drugs that affect the evaluation of therapeutic efficacy, such as unapproved chemotherapeutic drugs, targeted drugs, bio-immune agents, TCM, and other drugs that are clearly indicated in the instructions to be used for antitumor purposes, shall not be used in combination. On the other hand, detailed records should be made of the combined diseases or symptoms that existed before the start of the trial, and the drugs or other therapies that need to be continued or added must be recorded in the case report form with the generic name of the drug or the name of the other therapies, the dosage, the reason for use, the number of times of use, and the time of use, etc, so that they can be analyzed and reported at the time of the summary.

#### Provisions for Posttrial Care

After the clinical trial ends, if patients still require outpatient or inpatient care, they will continue to be seen by a physician. Any injuries that occur during participation will be covered by the trial insurance.

### Outcomes

#### The Primary Efficacy Endpoint

The primary efficacy endpoint is DFS. Refers to the time between the start of randomization and disease recurrence or death (from any cause). Imaging was performed using chest CT examination before the start of treatment and every 6 months after surgery. Patients found to have recurrent metastases were discharged from the group for standardized treatment when the pathological diagnosis was clear. Observation and follow-up until 5 years after surgery, DFS, and median survival calculation.

#### The Secondary Efficacy Endpoints

The secondary efficacy endpoints include OS, MRD, circulating tumor cell assay, Chinese medicine symptom score, quality-of-life assessment, immune indicators, tumor markers, SII, and PNI.

##### OS

Endpoint indicator, defined as the time from the start of randomization to death due to any cause. Overall survival and median survival calculations were performed with observational follow-up for up to 5 years postoperatively.

##### MRD

The second-generation sequencing (next-generation sequencing) method is used to detect MRD in the peripheral blood of the study participants to obtain a superior population screening model for TCM treatment.

##### Circulating Tumor Cell Assay

Circulating tumor cells will be detected once before and once after the intervention.

##### Specimen Collection Method

A total of 10 mL of venous blood was taken from the patient's median elbow vein with an ethylenediaminetetraacetic acid blood collection tube. The blood collection tube was repeatedly inverted and mixed, centrifuged at 2000g for 10 minutes, the supernatant was retained, and to further remove the redundant cellular components, it was centrifuged again at 8000 *g* for 10 minutes. Separated plasma was frozen and stored at –80 ℃ or extracted immediately according to the extraction kit instruction steps, respectively. The remaining blood cells should be added to saline to replenish 10ml, and then immediately isolate the circulating tumor cells.

##### Chinese Medicine Symptom Score

According to the “Shanghai TCM disease diagnosis and treatment routine,” 10 TCM symptoms of cough, sputum, chest pain, chest tightness, shortness of breath, fatigue, loss of appetite, insomnia, thirst, and spontaneous sweating were observed, and the severity was scored as 0-3, which was recorded once before and 6 months after the intervention.

##### Significant Effect

A reduction of the Chinese medicine symptom score greater than or equal to 70% after the intervention will be considered a significant effect.

##### Effective

A reduction of the Chinese medicine symptom score of greater than or equal to 30% and less than 70% after the intervention will render the treatment effective.

##### Ineffective

A reduction of the Chinese medicine symptom score of less than 30% after the intervention or an elevation in the score compared to the previous one will render the treatment ineffective.

##### Quality-of-Life Assessment

The quality-of-survival scale for patients with lung cancer EORTC QLQ-LC43 was used, which consists of EORTC QLQ-C30 (core scale) and EORTC QLQ-LC13 (the characteristic subscale of lung cancer). It mainly scores for patients with lung cancer on 5 domains related to functioning, general clinical symptoms, characteristic subsymptoms, general health status, and financial difficulties, and is recorded once before and 6 months after the intervention. The higher the score of function-related domains, the worse the function of the organism; the higher the score of general clinical symptoms and characteristic subsymptoms, the more serious the symptoms; the higher the score of general health status, the better the status; the higher the score of economic hardship status, the more difficult the economic hardship.

##### Immune Indicators

Cellular immunity (CD3, CD4, CD8, CD16, and CD56), regulatory T cells, myeloid-derived suppressor cells, natural killer cells, interleukin (IL)-1, IL-2, IL-6, IL-8, IL-10 Will be assessed once before and 6 months after intervention.

##### Tumor Markers

The following tumor markers will be assessed before and after treatment: CEA, CA125, CA153, Cyfra21-1, and SCC. At least 1 test will be performed before the intervention and 6 months after the intervention.

##### SII and PNI

The inflammatory response, immune status, and nutritional status of the body affect the tumor microenvironment and the prognosis of patients with tumors. Studies have shown that tumor-associated inflammation, especially host-associated systemic inflammation, is closely related to tumor development and progression, as well as the survival of cancer patients. SII is a comprehensive immune-inflammatory index, calculated based on the results of platelet, neutrophil, and lymphocyte counts. SII is a more objective predictor of the prognosis of patients with various types of tumors. It represents the balance between inflammation and immunity in patients with tumors. Inflammation, in turn, can influence tumor progression by affecting the host’s immunity and response to antitumor therapy. PNI is a nutritional prognostic indicator that can be used to reflect the nutritional and immune status of patients with tumors and is calculated from serum albumin and circulating lymphocyte counts. PNI is a nutritional prognostic indicator that can be used to reflect the nutritional and immune status of patients with tumors. Malnutrition is very common in patients with malignant tumors, especially advanced tumors. Day plays a crucial role in the process of tumor progression. There is also a relationship between tumor-associated inflammation and nutritional status. Studies have shown that tumor-secreted inflammatory mediators, including tumor necrosis factor and IL-6, lead to a loss of appetite and affect nutritional intake. Nutritional and immune status are associated with tumor progression and prognosis. The formula is as follows:

SII=peripheral blood platelet count (×10/L)×peripheral blood neutrophil count (×10/L)/peripheral blood lymphocyte count (×10%/L).

##### PNI

Serum albumin (g/L) + Lymphocyte count (10/L)×5.

Critical values were calculated based on receiver operating characteristic curves, and based on the critical values, the above indices were divided into high-level and low-level groups.

#### Baseline Data Observation

Demographic data such as gender, age, height, weight, and so on, were assessed. Vital signs such as blood pressure, heart rate, respiration, and so on were monitored. General clinical data included comorbidities and medications, and so on. Other data included information about education level, marital status, and so on.

#### Baseline Tumor Assessment and Risk Factors

Time of diagnosis, pathological type, degree of differentiation, tumor node metastasis classification of tumor, gene mutation status, comorbidities, surgical procedure, history of preoperative adjuvant therapy, smoking status, and family history of illness.

#### Safety Indicators

Blood routine analysis, liver and kidney function tests (including alanine aminotransferase, aspartate aminotransferase, glutamyl transpeptidase, blood creatinine, urea nitrogen, urinary protein, etc), electrocardiography, and monitoring of adverse events and serious adverse events (with detailed records at any time) Will be carried out once before the test, once every month during the treatment period, and once every 6 months during the follow-up period.

### Participant Timeline

Before the commencement of the trial, researchers must develop a study protocol and clearly define the participant timeline. The participant timeline for this study is presented in Table 3.

**Table 3:** Participant timeline for this trial.

Trial stage	Enrolment	Visiting period	Follow-up period	
	V^a^0	V1	V3	V4
Tine	D^b^0	6th month	12th month	18th month
Included or excluded cases	✓			
Sign the informed consent form	✓			
Randomization and blinding	✓			
Drug dispensing, drug recovery, and drug recording	✓			
Patient’s condition inquiry	✓			
Tumor pathology information	✓			
Western treatment modalities	✓	✓	✓	✓
Cancer pain assessment Scale	✓	✓	✓	✓
KPS score^c^	✓	✓	✓	✓
Chinese Medicine Symptoms Questionnaire（MDASI-TCM^d^）	✓	✓	✓	✓
PET-CT^e^ or CT examination	✓	✓	✓	✓
PFS^f^ overall survival	✓	✓	✓	✓
Tumor marker	✓	✓	✓	✓
Immunological indicators	✓	✓	✓	✓
Safety assessment	✓	✓	✓	✓
Adverse event records		✓		

^a^V: visit.

^b^D: day.

^c^KPS***:*** Karnofsky Performance Status.

^d^MDASI-TCM: MD Anderson Symptom Inventory for traditional Chinese medicine.

^e^PET-CT: Positron Emission Tomography-Computed Tomography.

^f^PFS: Postfinasteride syndrome.

### Compliance

We will record the number of medications returned by participants to calculate usage rates. We will take measures to improve adherence, including asking them to complete a statement as part of the informed consent process, emphasizing the importance of completing the study.

### Adverse Event Reporting and Harms

#### Safety Background Information Related to the Medicines Used in the Trial

According to the preclinical study, clinical trial information, and drug composition, it is necessary to pay attention to the observation of the changes in the hematopoietic system, digestive system, and urinary system of the patients during the trial, such as the number of red blood cells, hemoglobin content, blood creatinine and urea nitrogen, liver function, and so on.

#### Observation and Recording of Adverse Events

The investigator should carefully observe any adverse events occurring in the participants during the clinical study period and require the participants to truthfully reflect the changes in their condition after the administration of the drug, avoiding leading questions. While observing the efficacy, pay attention to the observation of adverse events or unanticipated toxic side effects (including symptoms, signs, and laboratory tests). Regardless of whether the adverse event is related to the test drug or not should be recorded in detail in the CRFs, including the time of the appearance of the adverse event, symptoms, signs, degree, duration, laboratory test indexes, treatment methods, after, results, follow-up time, etc, and should be recorded in detail in the case of the combined use of medication, in order to analyze the relevance of the adverse event to the test drug, the record should be signed and dated.

#### Medical Treatment of Participants

When adverse events are detected, the investigator may take necessary treatment measures according to the condition, such as adjusting the dosage, temporarily interrupting the medication, etc, and decide whether to terminate the trial. In the event of serious adverse events, the unit undertaking the trial study must immediately take the necessary treatment measures to protect the safety of the participants.

#### Judgment of Severity

Judgment of severity was based on the following criteria:

1. Mild: mild discomfort, the participant can tolerate, does not affect the treatment, does not need special treatment, no effect on the participant’s recovery.
2. Medium: moderate discomfort, intolerable to the participant, requiring special treatment, and having a direct impact on the participant’s recovery.
3. Severe: severe discomfort, endangering the life of the participant, death or disability, requiring immediate emergency treatment.

#### Judgment of Causal Relationship With Drugs

Indicators of causal judgement in the determination of adverse events include (1) whether the time of initiation of the drug is reasonably related to the appearance of the suspected adverse reaction; (2) whether the suspected adverse reaction corresponds to the type of adverse reaction known for the drug; (3) whether the suspected adverse reaction can be explained by the effect of the combination of the drugs, by the patient’s clinical condition, or by the effect of other therapies; (4) whether the suspected adverse reaction disappears or is alleviated by discontinuation or reduction of the dose; and (5) whether the suspected adverse reaction disappears or is alleviated after re-exposure to the drug. Whether the same reaction recurs after re-exposure to the suspected drug. Criteria for determining causality were established according to the above 5 judgment indicators in order (Table 4). Based on Table 4, we will determine the relationship between the following 5 levels of adverse events and the medicinal product, that are, 1: certainly related, 2: maybe related, 3: cannot be determined, 4: maybe not related, and 5: certainly not related. The incidence of adverse events was calculated using the total number of cases 1+2+3 as the numerator and all the enrolled cases available for adverse event evaluation as the denominator.

**Table 4:** Adverse event causation judgments.

Judgment results	Judgement indicators				
	1	2	3	4	5
Certainly relevant	Affirmative	Affirmative	Negative	Affirmative	Affirmative
Possibly relevant	Affirmative	Affirmative	Negative	Affirmative	Circumstances unknown
Undetermined	Affirmative	Affirmative	Difficult to affirm or deny	Difficult to affirm or deny	Circumstances unknown
Possibly irrelevant	Affirmative	Negative	Difficult to affirm or deny	Difficult to affirm or deny	Circumstances unknown
Definitely irrelevant	Negative	Negative	Affirmative	Negative	Negative

Based on the above table, the following 5 levels of adverse events were determined to be related to the drug that consists of 1: certainly related, 2: possibly related, 3: unable to determine, 4: possibly unrelated, and 5: certainly unrelated. The incidence of adverse reactions was calculated using the total number of cases 1+2+3 as the numerator and all the enrolled cases available for adverse reaction evaluation as the denominator.

#### Reporting and Handling of Adverse Events

The occurrence of any adverse event, such as the subjective discomfort of patients and abnormal laboratory tests, should be taken seriously, carefully analyzed, and immediate measures should be taken to protect the safety of the participants. Record in detail in the CRFs and retest in 24 hours and 7 days, and 14 days, as appropriate. Record its persistence, regression, and disappearance. Handling of serious adverse events: any serious adverse event that occurs during the trial must be reported immediately to the Medical Ethics Committee of the lead unit or the principal investigator by completing the Serious Adverse Event Report Form, and in the case of serious adverse reactions, to the State Food and Drug Administration within 24 hours. Notify the telephone number and contact person of the unit listed in the CRFs. When a patient has an emergency, the principal investigator of the research unit can open the emergency letter of the corresponding number taken by the patient (but there must be 2 witnesses present, and make corresponding records), according to the drug and the symptoms of the appropriate treatment, and notify the clinical monitor of the results of the treatment, and the researcher should be recorded in the case report form in detail on the reason for the breakthrough of blinding, date, treatment, results and sign. All adverse events should be followed up on until they are properly resolved or the condition is stabilized. The method of follow-up can be inpatient, outpatient, home visit, telephone, or newsletter, depending on the type and severity of the adverse event.

### Data Collection, Management, and Analysis

#### Data Collection Plans

All the original data in the case report form will be backed up and entered into the database by 2 people, proofread each other and correct the entry errors, and after checking the accuracy with the original data in the case report form, the data will be locked and then statistically analyzed. Construct a high-quality clinical research database, incorporate it into the unified management of the big data platform of the Oncology Research Center, and conduct long-term tracking and follow-up of the research participants.

#### Plans to Promote Participant Retention and Complete Follow‑Up

We will give each participant who cooperates in the participation and completion of the trial a traveling allowance of 100 CNY (equivalent to US $14.41, based on the exchange rate of US $1 to 6.9400 CNY on March 1, 2023) at the end of the treatment.

### Data Management

#### Establishment of Data Steering Committee

The subject leader, statisticians, and clinical scholars will work together to formulate standard treatment protocols, design trial protocols, organize the formulation and approval of various standard operating procedures, organize the formulation and approval of research plans, approve and decide whether to continue the trial, as well as approve the dissemination of trial results and write reports on the results.

#### Establishment of a Data Safety and Monitoring Board: Confidentiality

Investigators (clinicians, not involved in the inclusion of participants) and statisticians were responsible for designing the CRF, assessing the adequacy of the participant recruitment process, evaluating data quality, outcome safety assessment, outcome and effect assessment, recommending continuation of the trial, and reviewing and approving the dissemination of the results and the draft report. A full-time database administrator was responsible for the management of the database; an ombudsman was responsible for the monitoring of the data, and 2 data entry clerk responsible for data entry.

#### Data Capture and Database Management

Accurately collect and record process management information, data, and resultant measurement indicators and inspection data of observation items. A 2-person, 2-track entry into the electronic case information system is used, and after the entry is completed, the double data are checked and logical consistency verified. The database uses electronic case information system for data management.

#### Data Security and Monitoring

Data monitoring before the start of the clinical trial is generally on-site verification; real-time online monitoring and online quality control of the electronic case information system during the trial; the final data monitoring is carried out after the completion of the trial data collection, and the external data materials are reviewed and accepted.

#### Database Locking

After the completion of the interim analysis of the clinical trial and the data monitoring at the end of the trial, report to the data safety and monitoring board, the subject leader, with a list of data monitoring, and lock the database after obtaining a written signature of approval.

#### Plans for Collection, Laboratory Evaluation, and Storage of Biological Specimens for Genetic or Molecular Analysis in This Trial or Future Use

There are no further genetic analyses or storage in repositories planned.

### Data Analysis

#### Statistical Methods for Primary and Secondary Outcomes

Statistical software such as R4.4.0, SPSS 25.0, and GraphPad Prism were used for data analysis and statistical graphing in this study, with a test level of α taken as 0.05 and a 2-sided test, giving point estimates and 95% CIs.

#### Case Data Collectively: Full Analysis Set

All cases that were randomized, took the study drug at least once, and had at least one postdose efficacy assessment constituted the full analysis set (FAS) population of the study. Missing data in the efficacy-related part of the FAS population will be supplemented using a carry-over of data from the previous observation. FAS will be used for all analysis. PPS, the criteria for the PPS and its population will be finalized in the data and will include at least the following criteria: (1) compliance with the enrolment criteria set out in the trial protocol, (2) completion of all scheduled visits, and (3) no medication or treatment used during the trial that could have affected the evaluation of efficacy. The PPS is a secondary population for the evaluation of efficacy in this study.

Measurement data obeying normal distribution were expressed as mean and SD, 2 independent samples *t* test was used for intergroup differences, and paired *t* test was used for pre- and posttreatment comparisons within the group; if the data as a whole did not obey the normal distribution of measurement data, the median (first quartile, third quartile) was used, Wilcoxon rank-sum test was used for intergroup differences, and paired samples rank-sum test was used for pre- and posttreatment comparisons within the group; count data were expressed as frequencies and numbers; and PPS was the population of this study. And test; count data were expressed as frequency (n), constitutive ratio (%), and rate (%), and differences between groups were tested by chi-square test (Fisher exact probability test was used if the chi-square test was not satisfied); grade data were expressed as frequency and constitutive ratio, and differences between groups were tested by the Wilcoxon rank-sum test for independent samples.

Survival data, DFS, and OS were obtained using the Kaplan-Meier method and by plotting Kaplan-Meier curves to provide a visually intuitive depiction of differences between treatment groups. The estimation of treatment effect will be expressed by the hazard ratio and its 95% CI estimated by a stratified COX model. The dataset included in the statistics is based on intention-to-treat analysis.

Effect sizes and 95% CIs to quantify changes (not relying solely on *P* values) will be reported. FDR correction to account for multiple comparisons within related endpoint families (eg, all cytokines analyzed together) will be applied.

#### Interim Analyses

This study was a double-blind trial, and no interim analyses are planned.

#### Methods for Additional Analyses (eg, subgroup Analyses)

Due to the diversity of chemotherapy regimens and the severity of TCM symptoms, confounding factors may be introduced, affecting the accuracy of the results. We further conducted subgroup analysis and sensitivity analysis.

Based on the stratified model, the following predefined confounding factors (Chinese medicine symptom score and chemotherapy regimen types) were mandatorily included as covariates. Sensitivity analyses were conducted for the stratification factors and predefined covariates.

##### Chemotherapy Regimen Stratification Analysis

For different chemotherapy regimens, the interaction term between treatment group and chemotherapy regimen will be tested in the Cox model for the primary endpoint (DFS; with an interaction *P* value threshold of 0.10). If a significant modifying effect is present, the hazard ratio and 95% CI will be reported separately for each stratum.

##### TCM Syndrome Subgroup Analysis

Based on baseline TCM syndrome scores, divide into high or low severity subgroups according to the median or clinical cutoff value, and describe the trend of efficacy under different syndrome states.

##### Sensitivity Analysis

(1) Exclude data from centers using mixed chemotherapy regimens to validate the robustness of the conclusions, and (2) all multivariate models will be forced to adjust for chemotherapy regimen and baseline Chinese medicine symptom score.

##### Multiple Testing Control

Predefined subgroup analyses were adjusted using the Bonferroni method to control the significance level (α=0.05/number of subgroups) and minimize type I errors.

#### Methods in Analysis to Handle Protocol Nonadherence and Any Statistical Methods to Handle Missing Data

For missing values in the independent variables, multiple interpolation methods are used to select the optimal dataset. If there is a large amount of missing data, exceeding 20%, interpolation is not recommended.

#### Plans to Give Access to the Full Protocol, Participant‑Level Data, and Statistical Code

The full protocol, as well as datasets used and analyzed during this study, can be made available by the corresponding author upon reasonable request.

#### Monitoring

In order to strengthen the management of this project and improve the efficiency and effectiveness of implementation, the following management system and departments are established by 5 subcenters, namely, Shanghai Municipal Hospital of Traditional Chinese Medicine, Shanghai University of Traditional Chinese Medicine; Shanghai Lung Hospital of Tongji University; Shanghai Chest Hospital, Shanghai Jiao Tong University School of Medicine; Shanghai General Hospital, Shanghai Jiao Tong University School of Medicine; Shanghai Hospital of Integrated Traditional Chinese and Western Medicine, Shanghai University of Traditional Chinese Medicine.

#### Project Management Office

A project management office will be set up with the project supporting unit as the core, regulations and management systems will be formulated, and the responsibility system of the project leader will be implemented. The project leader is responsible for the day-to-day management of the project office, top-level design, project organization and implementation, and coordination of the work of the participant groups. Scientific research assistants are set up to coordinate the daily affairs of the project with the project leader, inform the progress of the project implementation on a regular basis, arrange relevant meetings, undertake the annual summary, midterm assessment, project acceptance of the material collation work, and coordinate the data analysis, summary of the results, and publication of papers. Set up a financial assistant, responsible for the management and reasonable distribution of funds. Invite big data analysis companies and statistical companies to join the project management office, such as providing data platform construction services and statistical analysis services for each topic.

#### Management Promotion Team

A hospital management promotion team covering the heads of all functional departments of the hospital is set up to provide financial support, technical support, and logistical support for the center.

#### Expert Steering Group

The expert steering group is headed by the applicant, and its members include oncology experts in the field of lung cancer from Shanghai Municipal Hospital of Traditional Chinese Medicine, Shanghai University of Traditional Chinese Medicine; Shanghai Lung Hospital of Tongji University; Shanghai Chest Hospital, Shanghai Jiao Tong University School of Medicine; Shanghai General Hospital, Shanghai Jiao Tong University School of Medicine; Shanghai Hospital of Integrated Traditional Chinese and Western Medicine, Shanghai University of Traditional Chinese Medicine.. The expert steering group is also invited to be composed of clinical experts, methodology experts, and statistical experts, to validate the research proposal and to discuss and solve the major issues, such as key points and difficulties of the project research.

#### Project Implementation Team

The person in charge of each subcenter and the scientific research assistant, and the financial assistant shall form a project implementation team, implement a negative communication system for the project leader, and regularly report and discuss the progress of the project and related matters.

#### Subject Supervision Committee

The hospital research center will set up a subject supervision committee, which is responsible for supervising the whole process of the research, guaranteeing the authenticity and objectivity of the research data, and is also responsible for the safety of information. The supervision committee will independently summarize the experimental data; all the clinical research subject undertakings will be supervised by the Health and Welfare Commission and the Ethics Committee, so as to effectively safeguard the legitimate rights and interests of the test participants.

#### Frequency and Plans for Auditing Trial Conduct

The monitoring will be carried out by a supervisor from the Clinical Research Center of the Shanghai Hospital of Traditional Chinese Medicine, who will visit the Center on a regular basis, depending on patient recruitment. It is carried out in accordance with the clinical monitoring program. Regular visits will be made after every 10 patients are included.

#### Plans for Communicating Important Protocol Amendments to Relevant Parties (eg, Trial Participants and Ethical Committees)

Any substantive changes to the research process must be in writing and state the reason for the change. They will be submitted to the Ethics Committee and ClinicalTrials.gov and will be implemented only after approval.

### Dissemination Plans

The results of our study will be presented at relevant scientific conferences or magazines.

### Ethical Considerations

The trial will be conducted in accordance with Good Clinical Practice guidelines; it will be supervised and managed by the Ethics Committee of Shanghai Municipal Hospital of Traditional Chinese Medicine, Shanghai University of Traditional Chinese Medicine. The trial was registered at ClinicalTrials.gov (NCT06381960), and the investigational plan was approved by Shanghai Municipal Hospital of Traditional Chinese Medicine, Shanghai University of Traditional Chinese Medicine (approval number 2023SHL-KY-19-01). All participants would sign the informed consent before joining the trial.

## Results

This research began receiving funding in October 2022. This study has been prospectively registered on ClinicalTrials.gov with the registration number NCT06381960. This study began screening and recruitment in March 2023. Recruitment is currently underway; by the end of 2024, a total of 180 eligible participants will be enrolled. Recruitment will continue until the end of June 2025 or until the target sample is reached. We estimate that the results will be published by March 2026.

## Discussion

In this trial, we hypothesize that the treatment group will have better efficacy and safety than the control group.

Lung cancer is the leading malignant tumor in China in terms of deaths, and metastasis is the key cause. For patients with lung cancer who have undergone radical surgery, there is currently a lack of effective chemotherapy regimens. This poses a dual challenge for this special patient group in clinical practice, as it not only limits the overall improvement in lung cancer treatment outcomes but also results in significant financial burdens on society and families due to the high costs of medical care.

With the development of molecular targeted therapy and immunotherapy, patients with intermediate and advanced lung cancer who are positive for driver genes or have high PD-L1 expression can further benefit from targeted therapy or immunotherapy. Adenocarcinoma and squamous carcinoma are common pathologies in lung cancer. A study that included 371 Chinese lung cancer cases showed that the mutation types in lung adenocarcinoma were Epidermal Growth Factor Receptor (EGFR; 60%), Tumor Protein P53 (57%), Kirsten Rat Sarcoma Viral Oncogene Homolog (13%), Phosphatidylinositol-4,5-Bisphosphate 3-Kinase Catalytic Subunit Alpha (7%), and Anaplastic Lymphoma Kinase (7%), whereas those in squamous lung carcinoma included Tumor Protein P53 (87%), Phosphatidylinositol-4,5-Bisphosphate 3-Kinase Catalytic Subunit Alpha (43%), Cyclin-Dependent Kinase Inhibitor 2A (20%), Lysine Methyltransferase 2D (20%), and EGFR (17%) mutations [9]. For Anaplastic Lymphoma Kinase /EGFR/Kirsten Rat Sarcoma Viral Oncogene Homolog /ROS Proto-Oncogene 1/MET Proto-Oncogene mutations, there are several targeted therapeutic agents have been approved, marketed internationally, and included in the health insurance. Wu Yilong et al conducted a clinical study confirming the benefit of ositinib in patients after radical surgery for stage IB-IIIA lung cancer with driver gene EGFR positivity [12,13]. Meanwhile, immunotherapy sequential treatment after chemotherapy for the Radical resection of lung cancer patients with stage IB-IIIA lung cancer with high PD-1/L1 expression (≥1%) may also provide a survival benefit and reduce the rate of metastasis. In addition, a study included 328 patients after resection of stage IA-IIB NSCLC to examine their immune checkpoint expression [14]. The study showed that 62.0% (160/258) of the lung adenocarcinoma group had PD-L1 <1%, and only 38.0% (98/258) had PD-L1 ≥1%, suggesting that the expression of immune checkpoints is low in lung adenocarcinoma patients [15]. Therefore, for patients with positive driver gene and high immune checkpoint expression after radical lung cancer surgery, standardized diagnostic and treatment protocols that can benefit patients clinically are already available. However, for patients with negative gene expression and low PD-1/L1 expression (1%) after radical lung adenocarcinoma surgery, who have poor chemotherapeutic efficacy in the clinic and lack indications for molecularly-targeted or immunotherapeutic treatments, the development of effective, scalable, and specific therapeutic protocols is urgently needed.

In summary, the existing multidisciplinary treatments available for lung cancer metastasis, such as chemotherapy, targeted therapy, and immunotherapy, have been developed based on alterations to the genetic structure and function of the tumor cells themselves in the primary foci. However, as tumor metastasis is often trans-organ and involves imbalances in the function of multiple systems, the overall efficiency of current strategies for the prevention and treatment of lung cancer metastasis is poor. TCM, which stresses the holistic concept and dialectical treatment, and commits to a people-centered approach, has unique advantages in preventing and treating tumor metastasis [15-18]. A large number of basic and clinical studies have confirmed that TCM can significantly inhibit the metastasis of lung cancer, significantly prolong the survival period of patients, and improve their quality of life [19,20]. Therefore, it is an important breakthrough to overcome the bottleneck of tumor metastasis in the clinic under the holistic view and syndrome differentiation of TCM to understand the pathogenesis of lung cancer metastasis from a systemic perspective, and then anchoring the key therapeutic targets to develop effective prevention and treatment strategies [21-23].

This research program inherits the theoretical advantages of Chinese medicine's “the Holistic Concept” and the thought of “Precentive Treatment of Disease,” inherits the academic thought of “Treating Cancer by Reforcing Healthy Qi,” and gives full play to the advantages of systems biology, precision medicine, and Chinese medicine. Preliminary research indicates that the method of tonifying the body and expelling pathogens can effectively inhibit lung cancer recurrence. In addition to Fuzheng Quxie Formula, the TCM formulations currently available for the treatment of NSCLC include Jinfukang Oral Liquid, Zilongjin Tablets, and Huachansu Oral Liquid. Compared to other TCM formulations, Fuzheng Quxie Formula is based on the principle of “supporting vital qi and dispelling hidden toxin” (supporting vital qi: enhancing immunity; dispelling hidden toxin: cytotoxicity). Its advantages are primarily reflected in the following aspects, such as (1) precise immune regulation involves simultaneously enhancing effector cells and inhibiting immune-suppressive cells; (2) extending survival and delaying resistance to targeted therapy, Fuzheng Quxie Formula is currently the only TCM compound clinically validated to delay resistance to EGFR- Tyrosine Kinase Inhibitor; (3) reducing toxicity and enhancing efficacy, improving tolerance to chemotherapy; (4) comprehensive coverage across treatment stages, from postoperative prevention of recurrence to late-stage targeted maintenance therapy, aligning with the trend toward personalized treatment for lung cancer. Comparison of key indicators between Fuzheng Quxie Formula and other Chinese herbal formulas are present in Table 5.

**Table 5:** Comparison of key indicators between Fuzheng Quxie Formula and other Chinese herbal formulas.

Indicators	Fuzheng Quxie Formula	Jinfukang oral liquid	Zilongjin tablets	Huachansu oral liquid
Efficacy	supporting vital qi and dispelling hidden toxins	Tonifying qi and nourishing yin	Tonifying qi and blood	Clear heat and detoxify, broad-spectrum anticancer
Prolong survival	Median OS^a^ extended by 5 months↑, PFS 11.8 months	Disease control rate: 71.2%	Survival time extended by 9.5 months	Improved quality of life, no clear OS data
Advantages of a combination targeted therapy	Reduce the risk of TKI^b^ resistance (HR^c^ = 0.888)	Unreported	Unreported	Unreported
Applicable stage	Late-stage TKI combination therapy or chemotherapy maintenance, or post surgery	Qi and Yin deficiency type lung cancer (alone or in combination with radiotherapy and chemotherapy)	Maintenance treatment for qi and blood deficiency	Adjuvant chemotherapy or palliative care for advanced disease

^a^OS: overall survival.

^b^TKI: Tyrosine Kinase Inhibitor.

^c^HR: hazard ratio.

Based on the theory of “Deficiency of Vital Qi and Hidden Toxin,” the research target is located in patients with stage IIA-IIIA lung cancer who are negative for the expression of driver genes after radical surgery, and carry out a multicenter clinical study on the combination of chemotherapy with Chinese medicine to prevent and control the recurrence and metastasis of IIA-IIIA stage IIA-IIIA driver gene-negative NSCLC after radical surgery. The study clarified the comprehensive efficacy of TCM in regulating the internal environment of the body’s immune function state to reduce recurrence and metastasis, with a view to obtaining high-level evidence-based medical evidence and forming a standardized diagnostic and treatment protocol of TCM for preventing and treating recurrence and metastasis of postoperative cancer that can be promoted and applied, so as to promote the improvement of the therapeutic efficacy of postradical treatment for patients with lung cancer. Second, based on the results of the study, the advantageous population of TCM for the prevention of recurrence and metastasis was screened out by combined MRD detection, and histopathology, circulating tumor cells, tumor-associated exosomes, immune indexes and other related indexes of postoperative patients with stage IIA-IIIA lung adenocarcinoma who were negative for the expression of driver genes were detected, and the therapeutic efficacy prediction model of postoperative patients with stage IIA-IIIA lung adenocarcinoma who were negative for the expression of driver genes was jointly established, so as to improve the precise treatment level of lung cancer after radical surgery and promote the overall prevention and control efficiency of lung cancer.

## Appendix

Multimedia Appendix 1 CONSORT checklist.

## Abbreviations

CRF: case report forms
DFS: disease-free survival
EGFR: epidermal growth factor receptor
FAS: full analysis set
IL: interleukin
MRD: minimal residual disease
NSCLC: non–small cell lung cancer
OS: overall survival
PD-1: programed death-1
PD-L1: programmed death-1 ligand
PNI: prognostic nutritional index
PPS: compliance with the protocol set
SII: systemic immune-inflammation index
TCM: traditional Chinese medicine

## Data Availability

The datasets used and/or analyzed during the current study are available from the corresponding author on reasonable request.
